# Altered Topological Organization of Functional Brain Networks in Betel Quid Dependence: A Resting-State Functional MRI Study

**DOI:** 10.3389/fpsyt.2021.779878

**Published:** 2022-01-03

**Authors:** Tao Liu, Liting Liu, Hui Juan Chen, Qingqing Fu, Lili Fu, Weiyuan Huang, Feng Chen

**Affiliations:** ^1^Department of Neurology, Hainan General Hospital (Hainan Affiliated Hospital of Hainan Medical University), Haikou, China; ^2^Department of Radiology, Hainan General Hospital (Hainan Affiliated Hospital of Hainan Medical University), Haikou, China

**Keywords:** betel quid chewers, resting-state functional magnetic resonance imaging, brain networks, topological organization, small-worldness

## Abstract

**Background:** Betel quid dependence (BQD) is associated with abnormalities in the widespread inter-regional functional connectivity of the brain. However, no studies focused on the abnormalities in the topological organization of brain functional networks in chewers in Mainland China.

**Methods:** In the current study, resting-state functional magnetic resonance images were acquired from 53 BQD individuals and 37 gender- and age-matched healthy controls (HCs). A functional network was constructed by calculating the Pearson correlation coefficients among 90 subregions in the human Brainnetome Atlas. The topological parameters were compared between BQD individuals and HCs.

**Results:** The results showed that BQD individuals presented a small-world topology, but the normalized characteristic path length (λ) increased compared with HCs (0.563 ± 0.030 vs. 0.550 ± 0.027). Compared to HCs, BQ chewers showed increased betweenness centrality (Be) in the right supplementary motor area, right medial superior frontal gyrus, right paracentral lobule, right insula, left posterior cingulate gyrus, right hippocampus, right post-central gyrus, right superior parietal gyrus, and right supramarginal gyrus, while decreased Be was found in the orbitofrontal area and temporal area, which is associated with reward network, cognitive system, and default mode network. The area under the curve (AUC) value of λ displayed a positive correlation with the duration of BQ chewing (*r* = 0.410, *p* = 0.002).

**Conclusions:** The present study revealed the disruption of functional connectome in brain areas of BQD individuals. The findings may improve our understanding of the neural mechanism of BQD from a brain functional network topological organization perspective.

## Introduction

Globally, ~600 million people use betel quid (BQ), making it the fourth most common psychoactive drug (1). Regular, prolonged BQ utilization is related to several health issues, which mainly include oral submucous fibrosis and oral cancer (1, 2). The carcinogenic potential of BQ was recognized by the International Association of Cancer Research in Lyon (France) in 2004 (3). The World Health Organization has identified BQ as a class I carcinogen. Long-term use of BQ can also lead to diseases of the central and peripheral nervous systems, heart, lung, skeletal system, gastrointestinal tract, and reproductive organs (3). Therefore, it is urgent to uncover the addictive mechanism of BQ dependence (BQD) and develop corresponding strategies to decrease the potential dangers of BQ.

BQD and its symptoms including tolerance, impulsive drug seeking, and withdrawal symptoms have been assessed by the Diagnostic and Statistical Manual of Mental Disorders, fourth edition (DSM-IV) criteria (4) and some dependence scales for other substances. An umbrella term of betel quid use disorder (BUD) was recently used based on DSM-5 criteria for substance-use disorders (5). However, multiple reports applied the Betel Quid Dependence Scale (BQDS) for screening all subjects. BQDS, developed by Lee etc., is an initial instrument specifically for measuring BQD (6). It comprised three factors: “physical and psychological urgent need,” “increasing dose,” and “maladaptive use.” It was found to have good internal consistency (α = 0.92), construct validity, and more suitability for selecting BQD individuals (6, 7).

Alkaloids were the main active ingredients in BQ. Major alkaloids, such as arecoline, arecaidine, guavacine, and guavacoline, make up ~2% of the BQ composition. Among these alkaloids, arecoline is the most physiologically important chemical constituent. It acts as a monoamine oxidase-A (MAO-A) inhibitor and thereby causing a significant increase in serotonin (5-hydroxytryptamine, 5-HT) level, a neurotransmitter that makes people pleasant and impulsive when binds to 5-HT receptors, contributing to BQD (8). Evidence indicates that the 5-HT2C receptor (5-HT2CR) and 5-HT2AR co-localized in the same neurons within the medial prefrontal cortex (mPFC) regulate decision-making, executive function, reward-related memory, and learning process (9). In addition, arecoline also has the partial agonist effect on nicotinic acetylcholine receptors (nAChR) including α4^*^ and α6^*^, although the effect is 20–250 times less potent than equimolar nicotine (10). To date, the limited understanding of BQD hampers the development of efficient treatments (1). Thus, it is of importance to investigate the neural mechanism of BQD.

Resting-state functional MR imaging (rs-fMRI) provides a non-invasive method to explore the mechanism of neuropsychiatric diseases. Because of efficiency and repeatability, easy to standardize, and without interference of tasks, rs-fMRI has been applied in the investigation of BQ chewers (11–17). The alterations in BQ chewers in those studies mainly involved the activation of the reward system including the orbitofrontal, medial frontal/ACC, the midbrain, the ventral tegmental area and pons, caudate, and thalamus, and the weakening of inhibition control (PFC, orbital frontal cortex, and ACC). A decline in executive functions including emotions, cognition, and affective decision making (cerebellum, occipital/temporal, frontoparietal, occipital/parietal, and frontotemporal networks, the para hippocampal/hypothalamus and medial–frontal cortex, etc.) was also found in BQ chewers and BQD individuals (4).

Human brains encompass various functional regions that interact and coordinate with each other. Graph-based theoretical methods have helped non-invasively assess the brain topology (18). The intrinsic activity of the normal brain likely generates efficient “small-world” networks, with a good balance between local specialization and global integration to correctly process information (19). Small-world networks are characterized by high-level local clustering with short path lengths and reduced network cost (20). Unlike traditional approaches, the methods based on graph theory give insights into the overall connectivity patterns of brain regions. Graph theory methods have been successfully applied to explore the mechanism of addiction behavior and network integration disruptions. For example, declined efficiency was reported in alcohol dependence (21), heavy smokers (22), and internet gaming addiction (23). Chewing BQ produces enhanced concentration, mild euphoria, relaxation, and post-prandial satisfaction in order to improve work and social skills (24), which seem to be related to the improvement of the efficiency of brain function activities. A previous study found altered long- and short-range functional connectivity in individuals with BQD (11). However, the topological properties of the brain network of BQD are still not very clear, and further research is needed.

The present study aimed to investigate topological organization of BQD individuals without smoke. The associations between topological alterations and clinical indexes such as disease course and severity would also be examined. We hypothesized that aberrant global and nodal parameters of the topological organization might be found in the BQD individuals, which might be associated with BQD duration and severity.

## Materials and Methods

### Subjects

The present study had approval from the ethics review board of Hainan General Hospital (Haikou, China) based on the Declaration of Helsinki (2000). Signed informed consent was provided by each participant before inclusion.

BQD volunteers were recruited *via* advertisement. Individuals using BQ with no tobacco ≥1 time/day for 5 years or more were considered volunteer candidates for entering the BQD group. The BQDS was used to screen all subjects. BQD participants conformed to the criteria for present BQ addiction, as diagnosed by the total BQDS >4 (6). Cross-sectional studies of patient specimens have revealed that addiction often co-occurred with psychiatric disorders, especially affective (e.g., depression) and anxiety (social anxiety disorder and generalized anxiety disorder) disorders. To rule out the influences of depression and anxiety, the 14-item Hamilton Anxiety (HAMA-14) and 17-item Hamilton Depression (HAMD-17) Rating Scales (25) for self-report were applied for participant evaluation before MRI scanning on the same day. BQD was diagnosed as BQDS >4, HAMA-14 <7, and HAMD-17 <8. Individuals not using BQ, areca nut, or tobacco were included as the health control (HC) group.

Exclusion criteria for all participants were the following: (1) tobacco use; (2) usage of various smokeless tobacco products such as gutka and paan masala; (3) self-reported systemic diseases, including neurological issues, cardiovascular disease, diabetes, and thyroid and kidney diseases; (4) current or past history of Axis I psychiatric and/or substance use-related disorders as assessed by a semi-structured personal interview; (5) current utilization of psychotropic products; (6) left-handedness; and (7) inability to read and write Chinese.

A questionnaire in simplified Chinese was distributed to all participants. The obtained information includes age, gender, educational status, and medical history including psychiatric disorders, HAMA-14, HAMD-17, BQDS, the alcohol dosage in the past 30 days, and the duration of BQ chewing habit. This procedure took 11 months, from January 2018 to November 2018. At first, 65 BQD individuals and 45 controls were included for MRI scanning, but only 53 BQD volunteers and 37 control individuals were finally included in the study. Others were excluded because of arachnoid cyst, angiocavernoma, lacunar infarction, and excessive head movement, respectively.

### MRI Data Acquisition

MRI was performed on a Siemens Verio3T MRI scanner with a standard 32-channel head coil (Germany) at the Department of Radiology, Hainan General Hospital. The participants were instructed to keep eyes open but not to think of anything in particular and keep their head motionless. To exclude gross cerebral pathology, routine structural MRI scans were obtained. Afterwards, spin-echo imaging was employed to collect anatomical images of functional regions in the axial plan parallel to the anterior commissure–posterior commissure (AC–PC) line. Scanning for whole-brain function assessment was performed by T2^*^-weighted echo-planar imaging (EPI) with blood–oxygen-level-dependent imaging (BOLD) contrast sensitivity [repetition time, 2,000 ms; echo time, 30 ms; field of view (FOV), 240 mm × 240 mm; flip angle, 80°; image matrix, 64 × 64; voxel size, 3.5 mm × 3.5 mm × 3.5 mm; every brain volume comprised 32 axial slices, and individual functional runs had 240 volumes]. High-resolution T1-weighted structural imaging was performed employing a magnetization prepared rapid gradient echo (MPRAGE) sequence (repetition time, 2,300 ms; echo time, 2.9 ms; TI, 900 ms; FOV, 256 mm × 256 mm; flip angle, 9°; in-plane matrix, 256 × 256; slice thickness, 1 mm; no gap; voxel dimension, 1 mm × 1 mm × 1.33 mm).

### Functional Image Pre-processing

fMRI imaging data were pre-processed using Data Processing Assistant for Resting-State Functional MR Imaging (DPARSF; http://www.restfmri.net/forum/DPARSF) via Statistical Parametric Mapping 8 (SPM8; http://www.fil.ion.ucl.ac.uk/spm/) and the rs-fMRI data analysis toolkit (REST1.8; http://www.restfmri.net). The initial 10 volumes of individual functional time series were discarded. Slice timing and realignment were performed to correct for possible head motion. Participants with head motion >1.5 mm translation and/or >1.5° rotation in any direction were excluded. Spatial normalization was performed with the SPM8 package to standard coordinates and resampled to 3 × 3 × 3 mm^3^. Individual T1-weighted anatomical images were co-registered to the mean functional images with a linear transformation. By means of the unified segmentation algorithm, the transformed structural images were then segmented into white matter, gray matter, and cerebrospinal fluid and normalized spatially in the Montreal Neurological Institute (MNI) space. The segmentation algorithm is deemed to have the capability of addressing the circularity problems of registration and tissue classification in optimized voxel-based morphometry. Finally, image smoothing was carried out using a Gaussian kernel of 4 mm, and then, linear trend and band-pass filtering (0.01–0.08 Hz) were carried out to remove the effects of low-frequency drift and high-frequency noise.

### Functional Network Generation

First, whole brain partition into 90 anatomical regions of interest (ROIs) was performed according to the automated anatomical template (26). One ROI was considered as an individual node of the functional network. Then, the time series of individual nodes were averaged, and pairwise Pearson correlation coefficients of the mean time series of various region pairs were considered as the functional network's edges, resulting in 90 × 90 correlation matrixes for individual participants. The correlation matrixes of every participant were determined *via* time courses of the ROIs, and the obtained correlation values underwent conversion by Fisher Z-transformation. The absolute z values underwent conversion into binary, undirected connection matrixes for different individuals based on a preset threshold for generating a graphic model of the brain network. In this study, 10% (27) ≤ sparsity (S) ≤ 50% (14) (interval, 0.01) was applied as previously proposed: (1) the minimal value of S was the average degree over all nodes of any threshold network above 2 × log(N), where N is the node number (*N* = 90); (2) the maximum value of S was determined by the percent of connections present using the most lenient threshold applied, which was 0.5. The threshold range could properly assess the small-world network properties and minimize the number of spurious edges of each network (28).

### Graph Theoretical Analysis

Networks were assessed according to sparsity threshold S. GRETNA (http://www.nitrc.org/projects/gretna/). GraphVar (http://www.nitrc.org/projects/graphvar/) was employed for generating topological network metrics. Brain functional networks were visualized with BrainNet Viewer. Seven common global metrics were assessed as follows: clustering coefficient (Cp), characteristic path length (Lp), small worldness (σ), normalized clustering coefficient (γ), normalized characteristic path length (λ), global efficiency (Eglob), and local efficiency (Eloc). The area under the curve (AUC) over sparsity ranges between 0.05 and 0.5 providing a total scalar for the topographic parameters of brain networks was computed (29).

At the nodal level, efficiency including the nodal global efficiency (Ei-glob), the nodal local efficiency (Ei-loc), and betweenness centrality (Be) were determined (18). These network metrics have been previously detailed (30); see details in Table 1.

**Table 1:** Global and nodal metrics for graph theoretical analysis.

**Graph theoretical analysis**	**The meaning of the parameters**
**Global metrics**	
Clustering coefficient (Cp)	The average of all clustering coefficients in the network; reflecting the tightness of connections between nodes
Normalized clustering coefficient (γ)	γ = Cp/Crandom; reflecting the deviation of the network's Cp from those of surrogate random networks
Normalized characteristic path length (λ)	γ = Lp/Lrandom; reflecting the deviation of the network's Lp from those of surrogate random networks
Characteristic path length (Lp)	The average of all shortest path lengths in the network; evaluating the information transmission capacity of the network
Global efficiency (Eglob)	The average value of the global efficiency of all nodes in the network; assessing the network's capability of transmitting information globally; Eglob is the reciprocal of Lp; the shorter the Lp, the higher the efficiency of Eglo, and the faster the information transfer rate between nodes
Local efficiency (Eloc)	The average value of the local efficiency of all nodes in the network; evaluating the network's capability of transmitting information locally; reflecting the information exchange capability of the sub-network
Small worldness (σ)	Reflecting the extent of the network between randomness and order
Nodal metrics	
The nodal global efficiency (Ei_glob)	1/(N – 1) of the reciprocal sum of the shortest path length from a node to all other nodes in the network; reflecting node's capability of propagating information with other network's nodes
The nodal local efficiency (Ei_loc)	The average value of the global efficiency of the sub-network formed between other nodes directly connected to a node; representing the information transmission efficiency between the brain area and its neighboring
Betweenness centrality (Be)	Effects of individual nodes on information flow among the remaining network's nodes; reflecting the contribution rate of nodes in information exchange to other nodes

### Statistical Analysis

Statistical Product and Service Solutions (SPSS, USA) was employed for data analysis. Independent two-sample *t*-test and the chi-square test were performed for continuous data and proportions, respectively. *p* < 0.05 indicated statistical significance.

To confirm the significant between-group differences in network topological features and regional network data of topographic parameters, two-sample *t*-test was carried out using the Gretna software with age, gender, education levels, and head motion as covariates. AUC was determined to assess integrity for global network parameters, which was independent with single threshold value. In addition, the Bonferroni method was applied for multiple comparisons of nodal centralities at the nodal level. The significance level for group differences in regional network parameters was set at *p* < 0.05

In case of significant between-group difference in a network metric, partial correlation analysis was carried out to assess its associations with clinical parameters (disease course and BQDS) in individuals with BQD.

## Results

### Demographic Information

A total of 90 participants (53 BQD individuals and 37 controls) were included. Both groups were similar in gender distribution, age, and education level. The alcohol dosage in the past 30 days were 208.6 ± 36.8 g and 192.0 ± 35.4 g in BQD and HC group, respectively; therefore, there were no alcohol-caused effects on BQD usage. Ratings of depression and anxiety assessed by HAMA-14 and HAMD-17 did not reach the cutoff for clinical significance in BQD individuals and controls. The participants had chewed BQ with dependence for 14.1 ± 8.3 years (ranging between 5 and 30 years), with a BQDS of 8.8 ± 3.0 (range of 5–15). Table 2 summarizes the demographic features of both groups.

**Table 2:** Demographics and clinical characteristics of participants.

	**Betel quid dependence (BQD)**	**Healthy control (HC)**	**Statistics** **(***t*** or χ^2^)**	***p*** **-value**
Age	38.2 ± 11.0	41.9 ± 11.6	−1.551	0.125^a^
Gender (male/female)	37/16	24/13	0.244	0.621^b^
Education (years)	12.2 ± 2.8	12.9 ± 2.9	−1.412	0.158^a^
Betel Quid Dependence Scale (BQDS)	8.8 ± 3.0	N/A		
Duration of BQD (years)	14.1 ± 8.3	N/A		
Alcohol last 30 days (g)	208.6 ±36.8	192.0 ± 35.4	1.018	0.308
HAMA-14	1.6 ± 1.8	2.2 ± 1.9	−1.587	0.117^a^
HAMD-17	2.1± 2.2	2.6 ± 2.5	−1.080	0.283^a^

*Unless otherwise indicated, data are means ± standard deviations. Values are expressed as mean ± SD*.

*N/A, not applicable; HAMA-14, 14-item Hamilton Anxiety Rating Scale; HAMD-17, 17-item Hamilton Depression Rating Scale*.

a *The p-value for age, education, HAMA-14, and HAMD-17 difference between the two patient groups was obtained by independent-samples t-test*.

b *The p-value for gender distribution in the two groups was obtained by chi-square test*.

### Topological Parameters at Local and Global Level in BQD

**Figure 1:**
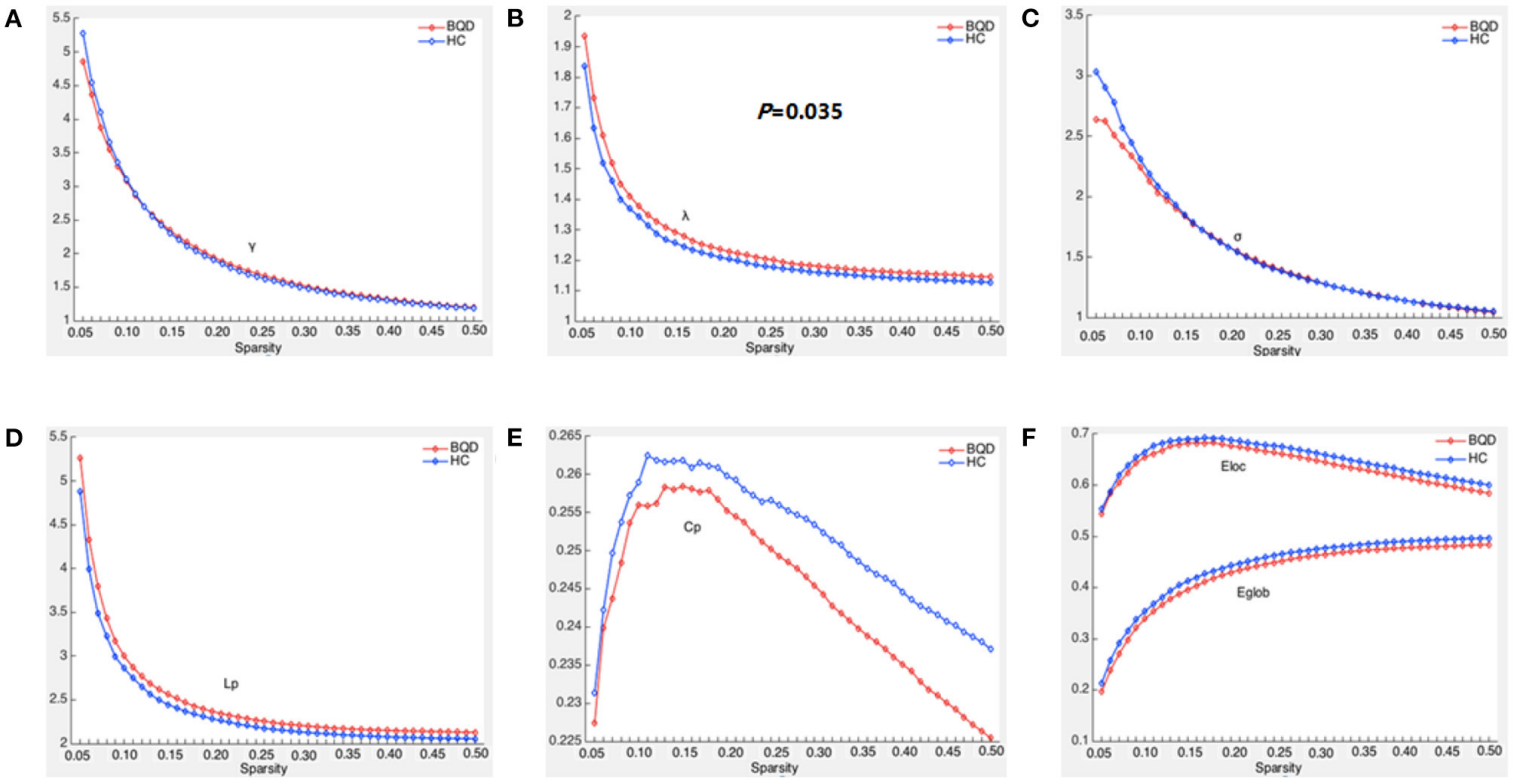
Global parameters of brain functional connectivity network in BQD group and HC group changed with threshold. **(A)** Within the threshold range, the normalized weighted clustering coefficient (γ) decreases with the increase of the threshold and is >1. **(B)** Within the threshold range, the normalized weighted characteristic shortest path length (λ) decreases with the increase in the threshold and is >1, and the λ parameter value of the BQD groups was larger than that of the HC groups. **(C)** Within the threshold range, the small world property (σ) decreases with the increase in the threshold and is >1. **(D)** Within the threshold range, weighted characteristic shortest path length (Lp) decreases with the increase in the threshold, and the Lp parameter value of the BQD groups was larger than that of the HC groups. **(E)** Within the threshold range, the weighted clustering coefficient (Cp) parameter value of the BQD groups was lower than that of the HC groups. **(F)** Within the threshold range, the global efficiency (Eglob) and local efficiency (Eloc) parameter value of the BQD group was lower than that of the HC group.

Within the set threshold of 0.05–0.5 and the step size set to 0.01, both BQD and HC groups had γ > 1, σ > 1, and λ ≈ 1, indicating that both groups showed typical small-world attributes for brain functional networks. The values of Lp, γ, λ, and σ are negatively correlated with the threshold in the defined range (Figure 1). Compared with the HC group, Lp and λ in the BQD group showed an upward tendency, while Cp, Eglob, and Eloc displayed a downward tendency at each threshold, also shown in Figure 1. However, no significant differences were found between the BQD group and the HC group in the AUC of γ (0.868 ± 0.149 and 0.866± 0.137, respectively), σ (0.682 ± 0.127 and 0.694 ± 0.116, respectively), Cp (0.110 ± 0.017 and 0.113 ± 0.015, respectively), Lp (1.108 ± 0.149 and 1.061 ± 0.115, respectively), Eglob (0.193 ± 0.028 and 0.199 ± 0.024, respectively), and Eloc (0.288 ± 0.049 and 0.293 ± 0.038, respectively), but λ increased significantly (0.563 ± 0.030 and 0.550 ± 0.027, respectively, *p* = 0.035) with age, gender, education levels, and head motion as covariates (Figure 1 and Table 3).

**Table 3:** Comparison of global parameters of the whole brain functional connectivity network between BQD group and HC group.

	**BQD**	**HC**	**t value**	***p*** **-value**
Cp	0.110 ± 0.017	0.113 ± 0.015	−0.938	0.351^b^
Lp	1.108 ± 0.149	1.061 ± 0.115	1.636	0.105^b^
γ	0.868 ± 0.149	0.866± 0.137	0.085	0.933^b^
λ	0.563 ± 0.030	0.550 ± 0.027	2.146	^*^0.035^b^
σ	0.682 ± 0.127	0.694 ± 0.116	−0.453	0.651^b^
E_glob_	0.193 ± 0.028	0.199 ± 0.024	−1.114	0.268^b^
E_loc_	0.288 ± 0.049	0.293 ± 0.038	−0.611	0.543^b^

*Unless otherwise indicated, data are means ± SD*.

b *The p-value between the two groups was obtained by independent sample t-test*.

* *The p < 0.05 between the two groups*.

*Cp, weighted clustering coefficient; Lp, weighted characteristic shortest path length; γ, normalized weighted clustering coefficient; λ, normalized weighted characteristic shortest path length; σ, small worldness properties; E_glob_, global efficiency; E_loc_, local efficiency; BQ, betel quid; HC, healthy control*.

### Altered Nodal Features in Brain Functional Networks in BQD

**Figure 2:**
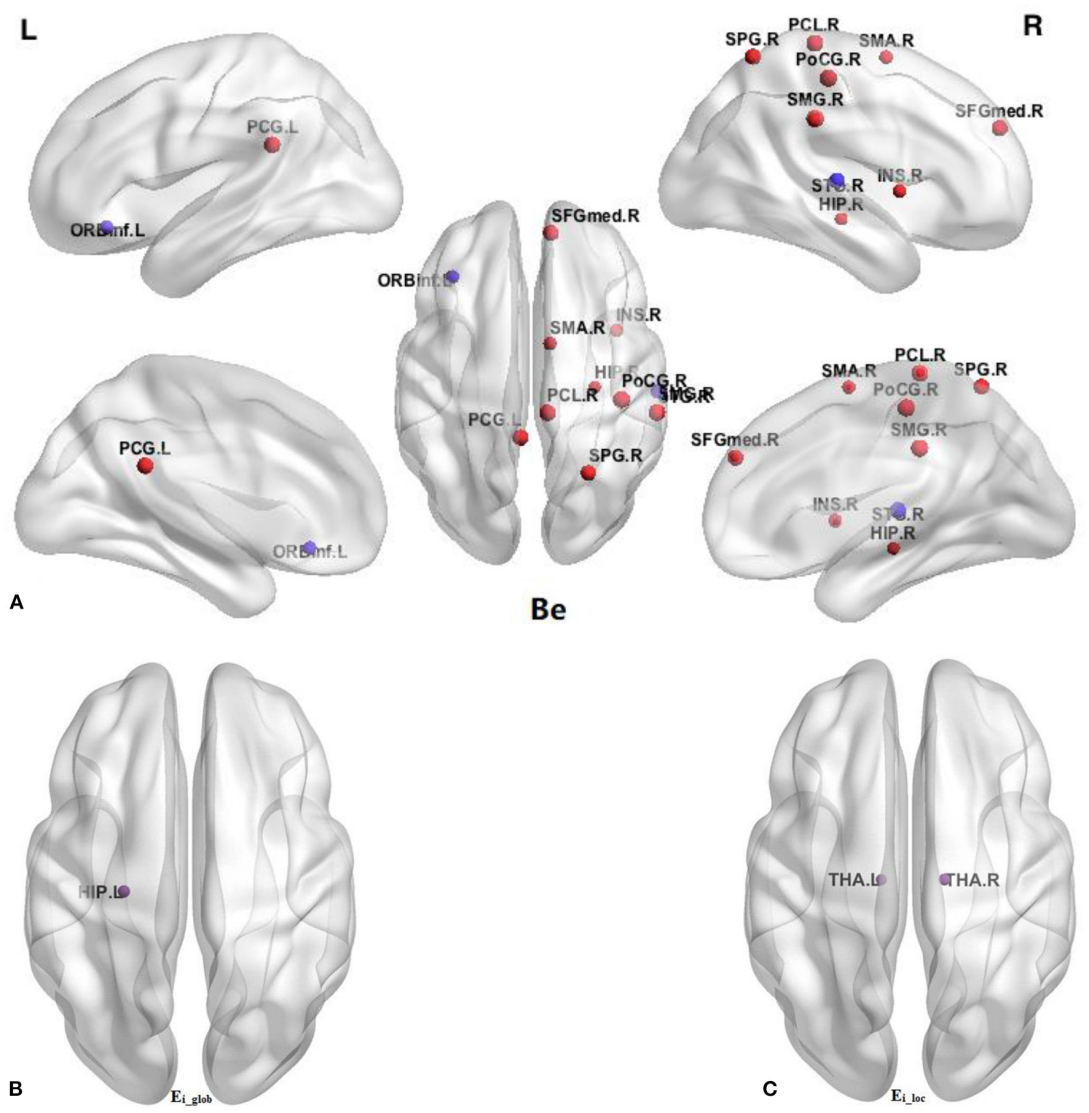
Comparison of nodal parameters of the whole brain functional connectivity network between BQD group and HC group. **(A)** Betweenness centrality (Be); **(B)** the nodal global efficiency (E_i_glob_); **(C)** the nodal local efficiency (E_i_loc_). The node size reflects the degree of attribute difference. Red indicates the increase in nodal parameters in the BQD group, while purple indicates the decrease in nodal parameters in the BQD group. ORBinf, orbital part of inferior frontal gyrus; SMA, supplementary motor area; SFGmed, medial superior frontal gyrus; INS, insula; PCG, posterior cingulate gyrus; HIP, hippocampus; PoCG, post-central gyrus; SPG, superior parietal gyrus; SMG, supramarginal gyrus; PCL, paracentral lobule; STG, superior temporal gyrus; THA, thalamus; L, left; R, right.

In comparison with the HC group, BQD individuals had markedly decreased Ei-glob in the left hippocampus (HIP.L) (0.138 ± 0.042 vs. 0.158 ± 0.039) and decreased Ei-loc in the left thalamus (THA.L) (0.182 ± 0.090 vs. 0.221 ± 0.084) and the right thalamus (THA.R) (0.174 ± 0.097 vs. 0.218 ± 0.088). Besides, decreased Be in BQD individuals were found in the orbital part of inferior frontal gyrus (ORBinf.R) (28.348 ± 18.877 vs. 37.853 ± 21.387) and right superior temporal gyrus (STG.R) (31.691 ± 24.994 vs. 48.738 ± 37.653). On the other hand, increased Be in the HC group were found (*p* < 0.05, Bonferroni correction) in the right supplementary motor area (SMA.R) (21.925 ± 18.102 vs. 14.397 ± 12.478), right medial superior frontal gyrus (SFGmed.R) (12.889 ± 12.480 vs. 7.063 ± 6.723), right insula (INS.R) (16.960 ± 22.363 vs. 8.107 ± 10.999), left posterior cingulate gyrus (PCG.L) (17.491 ± 16.272 vs. 9.883 ± 7.047), right hippocampus (HIP.R) (10.898 ± 14.636 vs. 5.526 ± 8.766), right post-central gyrus (PoCG.R) (18.382 ± 18.150 vs. 9.281 ± 9.244), right superior parietal gyrus (SPG.R) (12.101 ± 14.446 vs. 5.679 ± 5.893), right supramarginal gyrus (SMG.R) (19.356 ± 19.047 vs. 9.907 ± 9.127), and right paracentral lobule (PCL.R) (9.633 ± 14.559 vs. 4.241 ± 4.694). These results are demonstrated in Table 4 and Figure 2.

**Table 4:** Comparison of nodal parameters of the whole brain functional connectivity network between BQD group and HC group.

**Nodal parameters**	**Area**	**Brain region**	**BQD**	**HC**	**Relative difference of BQD to HC**	***t*** **-value**	***p-*** **value**
Betweenness centrality (Be)	15	ORBinf.L	28.348 ± 18.877	37.853 ± 21.387	↓	2.223	0.029
	20	SMA.R	21.925 ± 18.102	14.397 ± 12.478	↑	2.189	0.031
	24	SFGmed.R	12.889 ± 12.480	7.063 ± 6.723	↑	2.586	0.011
	30	INS.R	16.960 ± 22.363	8.107 ± 10.999	↑	2.209	0.030
	35	PCG.L	17.491 ± 16.272	9.883 ± 7.047	↑	2.670	0.009
	38	HIP.R	10.898 ± 14.636	5.526 ± 8.766	↑	2.031	0.045
	58	PoCG.R	18.382 ± 18.150	9.281 ± 9.244	↑	2.803	0.006
	60	SPG.R	12.101 ± 14.446	5.679 ± 5.893	↑	2.555	0.012
	64	SMG.R	19.356 ± 19.047	9.907 ± 9.127	↑	2.797	0.006
	70	PCL.R	9.633 ± 14.559	4.241 ± 4.694	↑	2.171	0.033
	82	STG.R	31.691 ± 24.994	48.738 ± 37.653	↓	2.581	0.012
The nodal global efficiency (Ei_glob)	37	HIP.L	0.138 ± 0.042	0.158 ± 0.039	↓	2.326	0.022
The nodal local efficiency (Ei_loc)	77	THA.L	0.182 ± 0.090	0.221 ± 0.084	↓	2.082	0.040
	78	THA.R	0.174 ± 0.097	0.218 ± 0.088	↓	2.218	0.029

*ORBinf, orbital part of inferior frontal gyrus; SMA, supplementary motor area; SFGmed, medial superior frontal gyrus; INS, insula; PCG, posterior cingulate gyrus; HIP, hippocampus; PoCG, post-central gyrus; SPG, superior parietal gyrus; SMG, supramarginal gyrus; PCL, paracentral lobule; STG, superior temporal gyrus; THA, thalamus; L, left; R, right*.

### Associations of Network Metrics With Clinical Parameters in BQD

The AUC value of λ displayed a positive correlation with duration of BQD (*r* = 0.410, *p* = 0.002), and no significant associations with BQDS (*r* = −0.211, *p* = 0.129) were found in BQD individuals. No significant associations were found in brain areas with altered nodal characteristic and clinical parameters s in BQD individuals.

## Discussion

The present study first investigated alterations in the topological organization of cerebral functional networks in BQD individuals without smoke by employing graph-based theoretical methods. The major findings included “normal appearing” small-world characteristics but significantly increased λ and abnormal nodal characteristics including decreased nodal efficiency in HIP.L and bilateral THA; increased Be in the SMA.R, SFGmed.R, PCL.R, INS.R, PCG.L, HIP.R, PoCG.R, SPG.R, and SMG.R; while decreased Be was found in the ORBinf.R and STG.R. The AUC value of λ displayed a positive correlation with the duration of BQD.

It is known that small-world networks have enhanced local clustering and short path length characteristics, ensuring effective information processing in a cost-effective manner (18, 31). As discussed previously, both BQD and HC groups showed the small-world feature (γ > l, λ ≈ l, σ >1), which was consistent with a previous research report (14), suggesting a balance between local specialization and global integration. As for common global network features (γ, σ, Cp, Lp, Eglob, and Eloc), BQD and HC groups were mostly similar, indicating a normal appearance of global integration in BQD individuals. However, different from the research of Weng et al. (14), the λ parameter was increased in BQD individuals, which may be attributed to the effect of the expanded sample size of our study and including women in our research. Some studies have found that gender differences can lead to a specific difference in the information transmission efficiency of the brain structure network. In general, women's brain function networks are more efficient than men (32). The γ and λ parameters reflect the relationship between actual networks and regular or random networks. Relative to the parameter γ, the λ parameter reflects the global information processing capacity of the functional brain network. Altered λ values indicate damaged global information processing ability of brain functional networks in BQD individuals, and this effect has a cumulative effect as the course of the disease increases. Even though the brain functional network in BQD individuals still has the small-world property, the optimized information processing ability related to “small-world” network may be altered. Compared with random networks, the speed of information dissemination and the ability of synchronization between long-distance areas in brain networks would decline, which may in turn decrease the specificity with which different neural structures control specific processing functions (33). Further research should be done to confirm the results.

In general, brain functional alternations contribute alternative to reward system enhancement and to declining inhibitory control that exercises control over reward-related behavior and executive functions in BQD (4). The nodal network metrics assessed in the whole brain indicated that dysfunctional integrations occur in the brain of resting-state BQD in some regions of the reward network (34) including a decrease in Ei-loc in bilateral THA, decrease in Be in SFGmed.R, and increase in Be in ORBinf.L, the latter two areas located in dlPFC. Be evaluated approximately how much information in the sub-network would pass through node i, which reflected the contribution rate of this node to other nodes in information exchange. The higher the Be value, the more prominent the position of the node in information exchange. Ei-loc and Ei-glob measure the ability of nodes to transmit information in the network. The thalamus is scarcely studied in association with addiction. Nevertheless, it is increasingly implicated in addiction because of its integrative function of controlling arousal and attention. For example, thalamic dopamine neurotransmission is enhanced after intravenous treatment with a stimulant medicine in cocaine users but not in control participants, as an effect associated with craving (35). Both PET and fMRI data demonstrated that thalamic activation is enhanced by primary and secondary rewards (vs. non-rewards) (36). Greater activation of the thalamus was observed in heavy episodic drinking participants, and it was positively correlated with alcohol intake and alcohol-related harmful consequences (37). In addition, the OFC and dlPFC may contribute to reward processing (38), which explains decision-making and goal-driven behavior anomalies in BQD (12). Based on previous reports and the above data, we hypothesized that the alterations of nodal indexes in reward networks might be at least partially associated with cognitive management and behavioral abnormalities driven by goal in BQD, but the direction of node parameter changes requires further research.

Our results also showed increased Be in INS.R, HIP.R, PoCG.R, and SPG.R, which might be related to cognitive impairment in BQ users, suggesting more efficient information transfer and integration in the cognitive network. This may be associated with impulsive drug-seeking behavior in BQD. BQ neuroimaging revealed enhanced connectivity in the frontoparietal, frontotemporal, occipital/parietal, and occipital/temporal/cerebellar regions (15, 17). Regular BQ users experience elevated alertness, euphoria, relaxation, arousal, enhanced motor responses, and a feeling of well-being (39), indicating that augmented connectivity might increase cognitive ability (17), thereby perpetuating addiction behavior. Interestingly, our results also showed decreased Be in STG.R and decreased Ei-glob in HIP.L. It was demonstrated that first exposure to stimulant drugs (e.g., cocaine, nicotine, and amphetamine) and alcohol might increase temporal function, with the generation of enhanced drug–context associations, which promote addiction development. Corroborating the self-medication hypothesis, withdrawal from stimulant drugs, alcohol, and cannabis impairs hippocampus-driven learning and memory, suggesting that alleviating such deficits might promote drug use relapse and maintain addiction (40). Systematically examining the function of the hippocampus in BQD might provide novel insights into addiction and related neural substrates.

Meanwhile, regions of the default mode network (DMN), which mainly involves PCG and SMG.R and SMA and paracentral lobule, showed increased Be in this study. DMN function is mostly linked to self-relevant, internally directed information processing (41). Our previous study reported that the overlaps of long- and short-range FCD in resting states were mainly found in brain areas comprising the DMN (42), highlighting the critical function of the DMN in both local and distant information processing by the brain in BQD. A fMRI study revealed that PCG, SMA, and inferior parietal cortex including SMG were also related to drug cues to elicit behavioral and psychophysiological reactions (43, 44). Although neuropsychological evaluation was not carried out here, we speculated that BQD would potentially affect both local and distant information processing in DMN. Neuromodulation may provide a new type of treatment for addiction since it can directly target abnormalities in neurocircuits (45).

Several limitations should be mentioned in the present study. First, this was a cross-sectional research; we cannot definitely assert the causation of the BQD and regional anomalies of topological organization. Therefore, longitudinal study is necessary to further clarify the cause and effect issues with an all-inclusive experimental design in future work. Second, we did not design elaborate neuropsychological measurements, so it was difficult to more accurately explain fMRI abnormal results. Third, further task-based fMRI investigation and neuropsychological testing are warranted to provide insights into higher-level functional alteration in chronic BQ users. Finally, BQD individuals without smoke were included in the study. BQD and smokers should be investigated in the future.

## Conclusions

In conclusion, the present study revealed regional anomalies of topological organization in the reward network, cognitive system, and DMN in BQD individuals without smoke utilizing graph-theory network assessment, especially in PCG.L, PoCG.R, and SMG.R. These results deepens our understanding of the BQD disorder.

## Data Availability Statement

The raw data supporting the conclusions of this article will be made available by the authors, without undue reservation.

## Ethics Statement

The studies involving human participants were reviewed and approved by the Ethics Review Board of Hainan General Hospital (Haikou, China). The patients/participants provided their written informed consent to participate in this study.

## Author Contributions

All authors listed have made a substantial, direct, and intellectual contribution to the work and approved it for publication.

## Funding

This work was supported by the National Nature Science Foundation of China (81760308 and 81971602 for FC, Grant No. 82160327 for TL, Grant No. 81871346 for WH) and Hainan Academician Innovation Platform Fund, Hainan Province Clinical Medical Center.

## Conflict of Interest

The authors declare that the research was conducted in the absence of any commercial or financial relationships that could be construed as a potential conflict of interest.

## Publisher's Note

All claims expressed in this article are solely those of the authors and do not necessarily represent those of their affiliated organizations, or those of the publisher, the editors and the reviewers. Any product that may be evaluated in this article, or claim that may be made by its manufacturer, is not guaranteed or endorsed by the publisher.
