# Corrigendum: Altered Topological Organization of Functional Brain Networks in Betel Quid Dependence: A Resting-State Functional MRI Study

**DOI:** 10.3389/fpsyt.2022.912951

**Published:** 2022-04-22

**Authors:** Tao Liu, Liting Liu, Hui Juan Chen, Qingqing Fu, Lili Fu, Weiyuan Huang, Feng Chen

**Affiliations:** ^1^Department of Neurology, Hainan General Hospital (Hainan Affiliated Hospital of Hainan Medical University), Haikou, China; ^2^Department of Radiology, Hainan General Hospital (Hainan Affiliated Hospital of Hainan Medical University), Haikou, China

**Keywords:** betel quid chewers, resting-state functional magnetic resonance imaging, brain networks, topological organization, small-worldness

In the original article, there was an error in the **Funding** statement. “Grant No. 8216070075” was incorrect, and has been changed to “Grant No. 82160327.”

The authors apologize for this error and state that this does not change the scientific conclusions of the article in any way. The original article has been updated.

## Publisher's Note

All claims expressed in this article are solely those of the authors and do not necessarily represent those of their affiliated organizations, or those of the publisher, the editors and the reviewers. Any product that may be evaluated in this article, or claim that may be made by its manufacturer, is not guaranteed or endorsed by the publisher.

